# Alveolar Ridge Reconstruction with Titanium Meshes and Simultaneous Implant Placement: A Retrospective, Multicenter Clinical Study

**DOI:** 10.1155/2016/5126838

**Published:** 2016-11-23

**Authors:** Raquel Zita Gomes, Andres Paraud Freixas, Chang-Hun Han, Sohueil Bechara, Isaac Tawil

**Affiliations:** ^1^Faculty of Dental Medicine, University of Oporto, Rua Manuel Pereira da Silva, 4200-393 Oporto, Portugal; ^2^Private Practice, Gamero #504, 2840941 Rancagua, Chile; ^3^EasyPlant Dental Clinic, Seo-Gu, Gwangju 4455, Republic of Korea; ^4^Department of Oral and Maxillofacial Surgery, Lithuanian University of Health Science, LT-44307 Kaunas, Lithuania; ^5^Private Practice, 345 Kings Highway, Brooklyn, NY 11223, USA

## Abstract

*Objective.* To evaluate horizontal bone gain and implant survival and complication rates in patients treated with titanium meshes placed simultaneously with dental implants and fixed over them.* Methods.* Twenty-five patients treated with 40 implants and simultaneous guided bone regeneration with titanium meshes (i–Gen®, MegaGen, Gyeongbuk, Republic of Korea) were selected for inclusion in the present retrospective multicenter study. Primary outcomes were horizontal bone gain and implant survival; secondary outcomes were biological and prosthetic complications.* Results.* After the removal of titanium meshes, the CBCT evaluation revealed a mean horizontal bone gain of 3.67 mm (±0.89). The most frequent complications were mild postoperative edema (12/25 patients: 48%) and discomfort after surgery (10/25 patients: 40%); these complications were resolved within one week. Titanium mesh exposure occurred in 6 patients (6/25 : 24%): one of these suffered partial loss of the graft and another experienced complete graft loss and implant failure. An implant survival rate of 97.5% (implant-based) and a peri-implant marginal bone loss of 0.43 mm (±0.15) were recorded after 1 year.* Conclusions*. The horizontal ridge reconstruction with titanium meshes placed simultaneously with dental implants achieved predictable satisfactory results. Prospective randomized controlled trials on a larger sample of patients are required to validate these positive outcomes.

## 1. Introduction

Dental implants are a predictable treatment procedure for the prosthetic rehabilitation of partially and fully edentulous patients [1–3].

An adequate bone volume is required for insertion of dental implants [4, 5]; the absence of a sufficient amount of horizontal and vertical bone is a problem that can affect the survival and success rates of dental implants in the short, medium, and long term [4, 5].

Since frequently patients present with bone defects of variable entity [4, 5], different surgical techniques have been proposed to restore the ideal anatomical conditions required for implant insertion or to allow simultaneously positioned implants to succeed [6–14]. These techniques include onlay/inlay bone grafting [6, 7], distraction osteogenesis [8], maxillary sinus augmentation [9], inferior alveolar nerve transposition [10], alveolar ridge split [11], and guided bone regeneration (GBR) with resorbable [12] and nonresorbable membranes, such as those in polytetrafluoroethylene (PTFE) [13] or titanium [14].

GBR is considered one of the most predictable of these techniques in terms of clinical outcomes, as reported by several systematic reviews of the literature [12–15], particularly where it is employed for the regeneration of defects of small and medium entities [16], or around dental implants [17]. The operating principle of GBR involves the placement of a mechanical barrier for the protection of the clot and the isolation of the bone defect from the surrounding connective tissues, in order to facilitate the selective recruitment of the mesenchymal cells responsible for new bone formation [12–15, 17]: this can allow the regeneration of the bone defect.

Bone regeneration with GBR has been demonstrated to be predictable, whether or not biomaterials are positioned below the membrane and are contained by it [12, 14, 16].

An ideal membrane should possess the following characteristics: biocompatibility, space maintenance capabilities, and ease of use [13, 14, 17, 18]. In the last few years, several types of membranes with different designs have been introduced, to facilitate the containment of the regenerative material that is often positioned below it and to prevent its dispersion, but also to simplify the work of the surgeon and the application of the membrane itself [13–18].

In particular, the titanium meshes represent a valid solution, because they meet most of the ideal requirements that a membrane should possess [14, 15]. Several clinical studies have demonstrated that titanium meshes can promote the formation of new bone, when positioned before [19–24] or simultaneously with dental implants [25–27].

The proper placement and stabilization of the titanium mesh into the defect site is of fundamental importance for the success of the regenerative therapy [13, 16–18]; one of the difficulties with these membranes can be related to this, particularly in case of simultaneous placement of the implant, for regeneration of small and medium size defects [17, 18, 25–27].

Recently, titanium meshes that can be fixed directly on the implant have been introduced, but there is still a lack of clinical studies evaluating the efficiency and predictability of these membranes [18, 26].

Therefore, the purpose of the present retrospective, multicenter clinical study is to evaluate the horizontal bone gain, the percentage of implant survival, and the degree of complications in patients treated with titanium meshes positioned simultaneously with dental implants and fixed over them.

## 2. Materials and Methods

### 2.1. Patient Selection

Patients enrolled in the present retrospective multicenter study were identified through the customized records of five different private dental clinics. Only records of patients with partial edentulism of the maxilla and/or mandible (with a period of edentulism of at least 4 months) or in need for replacement of nonrestorable failing teeth at the time of recruitment, who had been treated with titanium mesh and simultaneous implant placement in a period between January 2013 and December 2014, were reviewed. Further inclusion criteria for the present study were insufficient width of a portion of the alveolar process, with the need for horizontal augmentation of at least 3-4 mm, age > 18 years, good systemic and oral health, dentition in the opposing jaw, detailed information about the treatment, and a minimum follow-up of 1 year. In fact, the customized records of patients had to include all patient-related (gender, age at surgery, smoking habit, and history of periodontal disease) and implant-related (site, position, type of mesh used, type of prosthetic restoration, and date of provisional and definitive prosthesis delivery) information; in addition, they had to contain information about the occurrence of implant failures and/or biological and prosthetic complications during the entire follow-up period, since any complication that was manifested clinically was routinely referred back to the specialist practice for control. Exclusion criteria were any systemic disease that could contraindicate surgery (such as uncontrolled diabetes mellitus, immunocompromised status, coagulation disorders, radiotherapy, chemotherapy, alcohol or drug abuse, and use of oral and/or intravenous aminobisphosphonates), poor oral hygiene, and active periodontal infections. All patients had been informed about the planned treatment and had signed an informed consent form. All data were inserted into spreadsheet software and used for statistical evaluation. The study was performed in accordance with the principles outlined in the Helsinki Declaration on Human Experimentation, as revised in 2008.

### 2.2. Preoperative Work-Up

A preliminary clinical and radiographic examination had been performed prior to commencing the surgical procedures. All patients received a session of professional oral hygiene, with scaling and root planning, two weeks before surgery. In addition, patients were instructed about common oral hygiene procedures and were prescribed with chlorhexidine 0.2% mouthrinses, twice a day for 2 weeks, so that, before entering the surgical procedures, they all had an adequate plaque control. At the same time, a thorough radiographic examination was performed, in order to precisely assess the width of the (residual) alveolar process. Cone beam computed tomography (CBCT) scans were taken; then, raw CBCT data were imported into reconstruction software, where a careful three-dimensional (3D) evaluation of the alveolar process was performed. Linear and volumetric measurements were obtained, in order to fully disclose the anatomy of the bone site and therefore to choose the most appropriate implant and titanium mesh for reconstruction.

### 2.3. Dental Implants and Titanium Meshes

All patients were installed with tapered implants (AnyRidge®, MegaGen, Gyeongbuk, South Korea) characterised by strong self-cutting threads. These implants featured a 5 mm deep conical connection (10°) combined with an internal hexagon [28–30]. The aforementioned implants had a nanostructured calcium-incorporated surface [31]. The titanium membranes (i–Gen membranes, MegaGen, Gyeongbuk, South Korea) were available in 9 different configurations (type A for incisors/cuspids, type B for premolars, and type C for molars) with different size and shape (small, regular, or wide) in order to allow the clinician to graft all different sites (anterior and posterior sites) where a stable implant has been placed, but surrounding bone was insufficient. All these titanium meshes incorporated up to a 100° bend to provide adequate space for GBR. The titanium meshes had to be fixed on specially designed flat abutments (i–Gen screws, MegaGen, Gyeongbuk, South Korea) of variable height (1–3 mm), by means of a cover screw. The i–Gen kit included 12 titanium membranes, 6 i–Gen screws (flat abutments) for providing adequate space for regeneration, 6 cover screws for fixing the membrane to the flat abutments, and a hand hexagonal driver.

### 2.4. Surgical and Prosthetic Procedures

**Figure 1:**
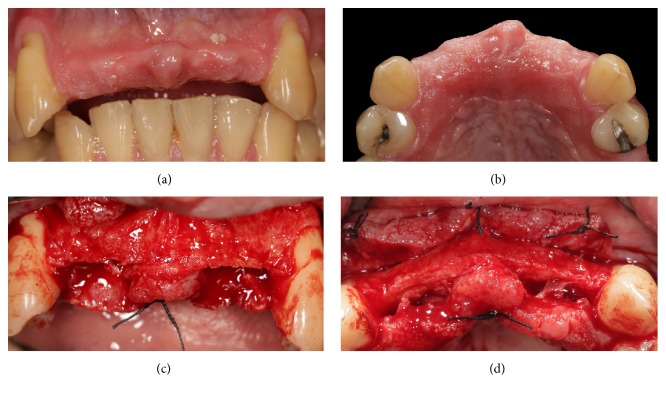
Presurgical clinical situation and elevation of full-thickness flap exposing the deficient alveolar ridge. (a) Preoperative clinical picture, frontal view; (b) preoperative clinical picture, occlusal view; (c) elevation of the mucoperiosteal flap, frontal view; (d) elevation of the mucoperiosteal flap, occlusal view.

**Figure 2:**
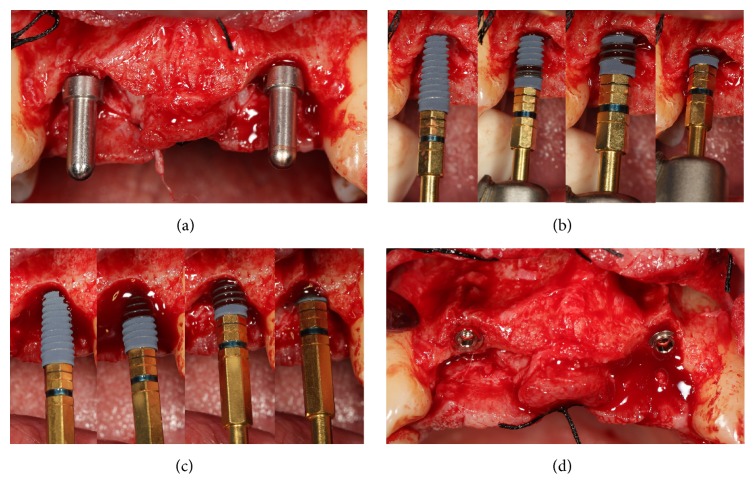
Preparation of the surgical sites and placement of the implants (AnyRidge, MegaGen, Gyeongbuk, Republic of Korea). (a) The implant sites have been prepared; (b) placement of the first implant in the position of the right lateral incisor; (c) placement of the second implant in the position of the left lateral incisor; (d) the implants* in situ*.

**Figure 3:**
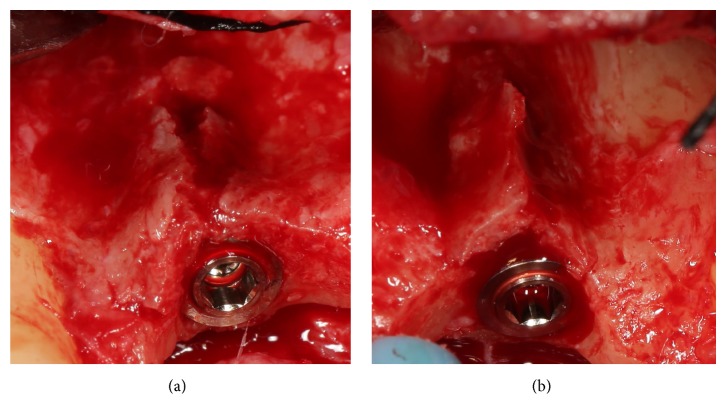
Details of the implant sites. (a) Details of the right implant site: fenestration of the thin buccal bone wall; (b) details of the left implant site: the buccal bone wall is thin and requires to be reinforced and protected.

**Figure 4:**
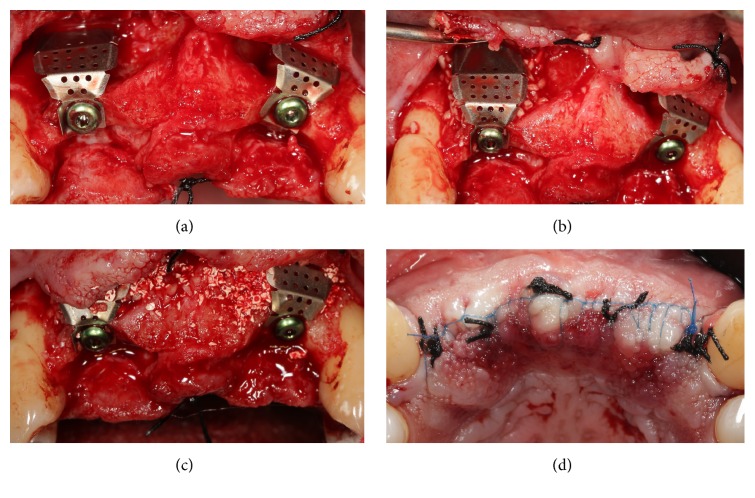
Placement of the titanium meshes (i–Gen, MegaGen, Gyeongbuk, Republic of Korea) and sutures. (a) The titanium meshes are connected to the implants and screwed on with the aid of a connecting screw; (b) particulate bone grafts are placed below the titanium mesh screwed on the right lateral incisor; (c) particulate bone grafts are placed below the titanium mesh screwed on the left lateral incisor; (d) sutures are performed.

**Figure 5:**
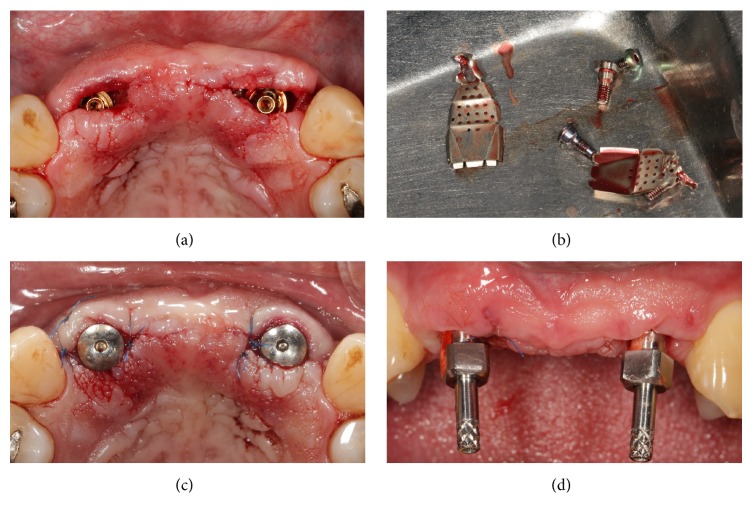
Second-stage surgery, removal of the titanium meshes and impressions. (a) and (b) Four months after placement, the titanium meshes were removed; (c) healing abutments were placed in position; (d) two weeks after placement of the healing abutments, impressions were taken.

**Figure 6:**
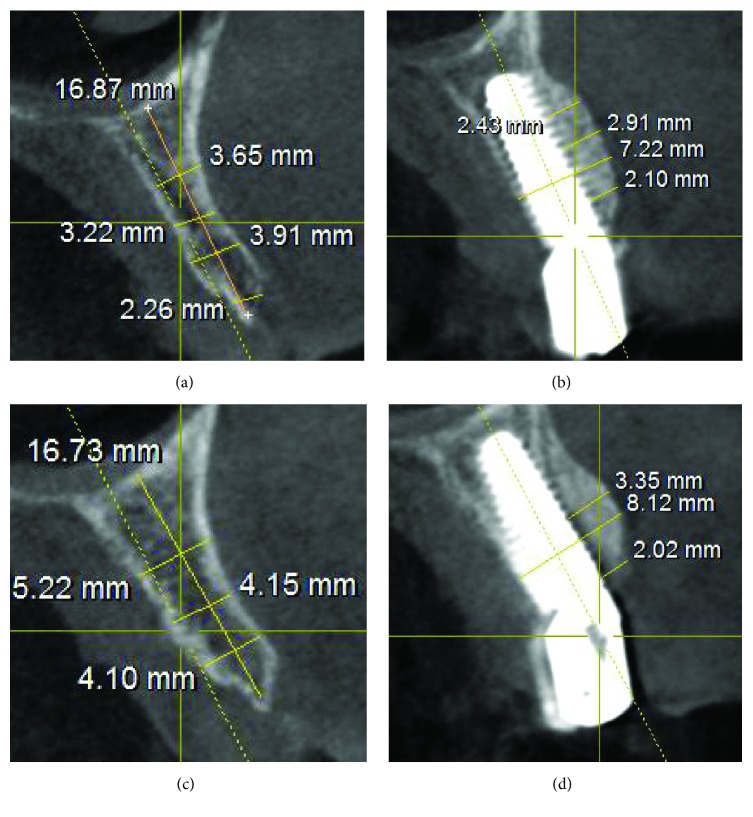
Cone beam computed tomography (CBCT) scans of the sites before surgery and after removal of the titanium meshes, three months later. (a) Right side: preoperative situation with a very thin residual alveolar ridge; (b) right side: the radiographic situation three months after surgery; (c) left side: preoperative situation with a thin residual alveolar ridge; (d) left side: the radiography three months after surgery.

**Figure 7:**
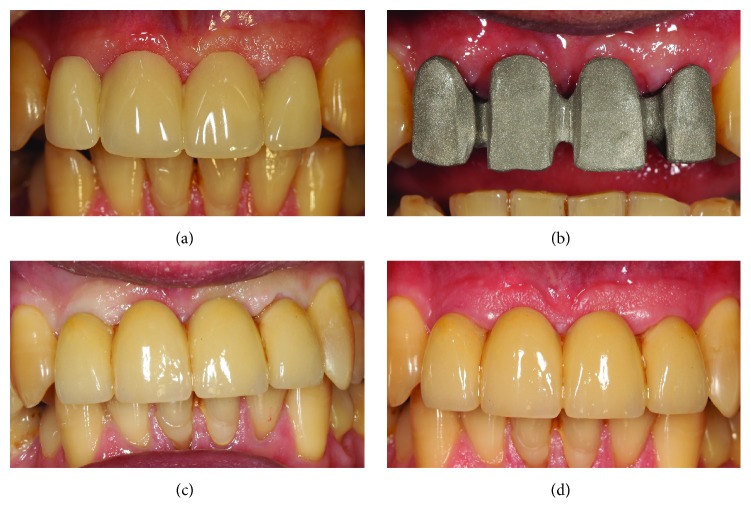
Prosthetic rehabilitations. (a) The provisional restoration* in situ*, two weeks after the first impressions; (b) three months later, the precision of final structure is tested clinically; (c) the application of the definitive metal-ceramic FPP; (d) the final FPP at the final control.

All the surgical and prosthetic procedures were performed under the same protocols, in the five different private clinical centers. After local anaesthesia, a paramarginal incision was made, connected with two wide releasing incisions. A full-thickness flap was raised to expose the residual bone and elevated on the buccal and palatal (lingual) aspect of the ridge (Figure 1); sutures were used for retraction. Several horizontal incisions were made in the periosteum, in order to widely mobilize the flap as far as possible, in the coronal direction. In the case of healed ridges, the surgeon proceeded with the osteotomy, starting with a 2.0 mm diameter pilot drill, to the desired depth. The preparation of the surgical site was based on the bone quality, using the set of helicoidal drills. After the preparation of the surgical sites, the implants were placed, slightly below the crestal level, using a hand ratchet (Figures 2 and 3). In patients with severely compromised dental elements, which called for extraction and immediate implant treatment, the teeth were gently extracted taking care not to further damage the remaining buccal bone wall. The alveolus was carefully cleaned in order to remove any granulation tissue. After irrigation with sterile saline, the integrity of the socket walls was checked. Once this was verified, the procedure continued with the preparation of the implant site. Once again, drill selection was based on the receiving site's bone quality; the implants were in a slightly subcrestal position, using a hand ratchet. For both healed and postextraction sites, there was not a specific threshold for insertion torque; the surgeon was free to decide the type of preparation and consequently the insertion torque. The stability of the implants was determined clinically as the absence of movement by the removal of the implant driver without use of the stabilizing wrench. After implant placement, the flat abutment of variable height (1–3 mm) was connected to the fixture, according to the clinical indications: a standard 1 mm cuff height was used in case of sufficient vertical space, but 2 or 3 mm cuff height could be chosen according to the situation. Then, the proper titanium membrane was selected, according to the size and shape of the bone defect. Each titanium membrane was adjusted to the individual anatomy and modelled in order to prepare the space for the regenerative material: these spaces were then filled with particulate bone grafts (Bio–Oss®, Geistlich Pharma AG, Wolhusen, Switzerland). The amount of material was sufficient to fill the space between the titanium meshes and the deficient buccal bone close to the fixtures (Figure 4). The titanium meshes were capable of maintaining the particulate bone* in situ*; then, they were fixed with a cover screw. An absorbable collagen membrane (Biomend® 15 × 20 mm, Zimmer Biomet, Warsaw, Ind, USA) could be adapted over the titanium meshes, according to the clinicians' preferences. The soft tissues were adapted over the membranes and care was taken in order to avoid tension during sutures. A tension-free closure was obtained through horizontal mattress sutures; single-loop sutures were made to further seal the incision line. Ice-packs were provided postoperatively, with the recommendation to keep them onto the treated area for at least 2 hours. Patients were prescribed oral antibiotics, amoxicillin plus clavulanic acid 1 gr every 12 hours, for 6 days. Postoperative pain was controlled by administering 600 mg ibuprofen every 12 h for 2 days. Patients were instructed to rinse with chlorhexidine digluconate 0.2%, 2-3 times per day, for an overall period of 2-3 weeks, with the recommendation to discontinue tooth brushing in the surgical area. A soft diet was recommended in this period, in order to avoid any trauma in the site of surgery; coherently, patients were asked not to wear removable dentures, where present, for a period of 1 month after surgery. Patients were recalled and checked at 2, 5, and 10 days after operation, to monitor their healing; 14 days after surgery, sutures were removed. After 3-4 months, a second-stage surgery was performed at the recipient sites. The fixtures were uncovered, and the titanium screws and meshes were removed (Figures 5 and 6); transmucosal healing abutments were positioned and sutures were performed around them. Two weeks later, impressions were taken, and temporary resin restorations (single crowns, SCs, and fixed partial prostheses, FPPs, either screw-retained or cemented) were provided. The temporary acrylic resin restorations were left for a period of 3 months, after which the definitive ceramometallic restorations were provided. All definitive restorations were ceramometallic, screwed, or cemented with temporary zinc oxide-eugenol cement (Figure 7). Before the delivery of the final restorations, occlusion was carefully checked. Maintenance care was provided every 6 months. All patients were controlled 1 year after the placement of the fixtures.

### 2.5. Primary Outcomes

#### 2.5.1. Horizontal Bone Gain

The horizontal dimensions of the alveolar ridge were measured in the CBCT sections, before and 4 months after the surgery, in mm. Basically, before implant placement, one first linear measure was taken at the future implant location; this measure was taken where CBCT evaluation revealed the maximum bone deficiency. After the second-stage surgery for the removal of the titanium mesh, the same measure was repeated at the same location. This second measure was registered; then the horizontal bone gain was determined by the difference between the second and the first measurement.

#### 2.5.2. Implant Survival

One year after implant placement, the prosthetic restorations were removed and the stability of all fixtures was verified. An implant was classified as “surviving” if still in function, without any problem, at the 1-year follow-up control. Conversely, failure to osseointegrate with implant mobility, progressive marginal bone loss due to bacterial tissue invasion (peri-implantitis), severe marginal bone loss in the absence of symptoms/signs of infection, and implant body fracture were the conditions in which implant removal was required.

### 2.6. Secondary Outcomes

#### 2.6.1. Early Biological Complications

Early complications were those that occurred immediately after surgery, or in the immediate aftermath (1-2 weeks), such as pain/discomfort, swelling/edema, and extraoral contusion.

#### 2.6.2. Late Biological Complications

All complications occurring from the third week after surgery, until the end of the study, were classified as late biological complications. These complications included titanium mesh exposure, partial or complete loss of the graft, and any disturbance in the function of the implant characterized by a biological process affecting the supporting tissues (peri-implant mucositis and peri-implantitis) and any peri-implant bone loss exceeding 1.5 mm, but in the absence of clinical signs of infection.

Peri-implant mucositis is the condition in which soft tissue inflammation, pain, and swelling are present, but in the absence of peri-implant bone loss; conversely, peri-implantitis is the condition in which pain, suppuration, exudation, and fistula formation are present, with concomitant probing pocket depth ≥6 mm and peri-implant marginal bone loss >2.5 mm.

The peri-implant marginal bone loss was calculated as previously reported [28–30]. In brief, intraoral peri-apical radiographs were taken at different times (at implant placement and 4 months and 1 year later, resp.) for each implant, using a rigid film-object X-ray source being coupled to a beam-aiming device (Rinn®; Dentsply, Elgin, IL, USA), in order to achieve reproducible exposure geometry. Customized polyvinyl-siloxane film holders were used to maintain the same angulation. Mesial and distal marginal bone levels of all implants were measured at different times with the aid of an ocular grid (4.5x magnification). The coronal margin of the implant neck and the most coronal bone-to-implant contact point were used as references for the linear measurements. To account for variability, the implant length was measured radiographically and compared with the actual dimensions; ratios were calculated to adjust for distortion. Peri-implant marginal bone loss was then calculated, as modification in the peri-implant marginal bone level at different time periods, on the mesial and distal implant side: the average from the mesial and distal calculations was used as the final value.

#### 2.6.3. Prosthetic Complications

All prosthetic complications that had affected the implant-supported restorations, from the placement of the provisional restorations and until the end of the study, were carefully registered. Mechanical complications included all complications occurring at prefabricated components (such as abutment screw loosening and abutment fracture) whereas technical complications included all complications of the laboratory-fabricated suprastructure or its materials (loss of retention, ceramic chipping, or fracture).

### 2.7. Statistical Analysis

Patient demographics and distribution of implants were analyzed using descriptive statistics.

Means and standard deviations as well as ranges and confidence intervals (95%) were calculated for quantitative variables, such as patient age, gain in horizontal dimensions of the alveolar ridge, and peri-implant marginal bone loss. Absolute and relative frequency distributions were calculated for qualitative variables, both patient-related (patient gender, age classes, smoking habit, and history of periodontal disease) and implant-related (implant site and position, surgical protocol, implant length and diameter, and type of prosthesis). The Chi-square test was used to evaluate the differences among the groups. The level of significance was set at 0.05. The incidence of biological complications (pain/discomfort and swelling/edema/extraoral contusion after surgery, membrane exposures and/or infection, graft loss, peri-implant mucositis, and peri-implantitis) and prosthetic complications (abutment screw loosening, abutment fracture, loss of retention, and ceramic chipping or fracture) as well as the implant survival rate were calculated, 1 year after implant placement. The implant survival rate was calculated both at the patient and at the implant level. All computations were carried out with dedicated statistical analysis software.

## 3. Results

In total, 25 patients (15 males, 10 females; aged between 43 and 69 years, mean age 54.3 ± 7.5) who had been treated with implant placement with simultaneous GBR with titanium meshes, were selected for the present retrospective, multicenter clinical study. The distribution of the patients is illustrated in Table 1. This distribution was uniform among the different groups, as no differences were found in the distribution by gender (*p* = 0.3173), age (*p* = 0.4677), or smoking habit (*p* = 0.0719); however, most of the patients had no history of periodontal disease (*p* = 0.0278). Forty implants were placed (32 in the maxilla and 8 in the mandible; 12 in anterior regions and 28 in posterior regions). Thirty-one implants were placed in healed sites, while 9 implants were installed in fresh extraction sockets. The distribution of the implants is shown in Table 2. There were significant differences in the distribution of the implants among the different groups. In fact, most of the implants were placed in the maxilla (*p* = 0.0001), were premolars (*p* = 0.0074), and were placed in healed ridges (*p* = 0.0005); the most frequently used implants were 10.0–11.5 mm in length (*p* = 0.0203) and the most frequent prosthetic restorations were SCs and short-span 2-unit FPPs (*p* = 0.0225). No differences were found in the distribution of the implants by diameter (0.0655). Forty titanium meshes were placed: 12 type A membranes (2 small, 7 regular, and 3 wide), 22 type B membranes (5 small, 13 regular, and 4 wide), and 6 type C membranes (2 small, 2 regular, and 2 wide). An absorbable collagen membrane was employed to protect the titanium meshes in 12 cases (12/25: 48%).

At the second-stage surgery and after the removal of the titanium meshes, the CBCT evaluation revealed a mean horizontal bone gain or augmentation of 3.67 mm (±0.89; median 3.6; CI 95%: 3.40–3.94). With regard to early biological complications, 10 patients (10/25: 40%) reported mild pain for the 3-4 days following surgery; however this discomfort was well tolerated with analgesics; conversely, 15 patients (15/25: 60%) had no discomfort or pain at all. Twelve patients (12/25: 48%) experienced mild postoperative edema; in 2 patients (2/25: 8%) this edema coexisted with extraoral contusion in the region. Eleven patients (11/25: 44%) had no edema at all. The mean time between implant placement and second-stage surgery (removal of the titanium meshes and placement of healing abutments) was 3.8 months. In most of the patients (19/25: 76%), healing proceeded without any delayed complication, and grafts appeared well incorporated into native bone. However, titanium mesh exposure occurred in 6 patients (6/25: 24%). In all these cases, weekly examinations were carried out, and mesh exposure was treated with a gentle cleaning of the area with an extra soft toothbrush soaked in chlorhexidine 1% gel. In addition, patients were asked to apply 1% chlorhexidine gel, 2 times per day, and were instructed to rinse with 0.12% chlorhexidine, 2-3 times per day. After this treatment, in 4 of these exposures, spontaneous coverage of the titanium membrane was found, with complete reepithelization of the areas and soft tissue closure, in a period between 3 and 4 weeks. These exposures did not prevent proper graft incorporation into native bone. In the remaining 2 cases, however, the titanium mesh had to be removed, because of nontreatable soft tissue defects followed by infection and loss of the graft. In one patient, the loss of the graft was partial, and it did not affect the survival of the implant; in the other one, however, the infection caused the complete loss of the graft and the implant. This implant failure was classified as “early failure,” because it occurred 2 months after surgery (before the connection of the prosthetic abutment) in a 45-year-old smoking female patient, without history of chronic periodontal disease. No other implant failures were reported. Among the restorations, 17 were SCs (17 implants), 6 were 2-unit FPPs (12 implants), 3 were 3-unit FPPs (6 implants), and 2 were 4-unit FPPs (4 implants), representing a total of 27 fixed partial prosthetic units available for analysis. No prosthetic complications were registered. All the 39 surviving implants were followed up for 1 year, for an overall survival rate of 97.5% (implant-based) and 96.0% (patient-based). The peri-implant marginal bone levels at the 1-year examination are reported in Table 3.

## 4. Discussion 

Several clinical studies [19–25] and systematic reviews [14, 15, 18] have documented the predictability of titanium meshes in supporting horizontal and vertical guided bone regeneration.

However, only a few of these studies [25–27] reported on alveolar ridge reconstruction with titanium meshes and simultaneous implant placement.

Von Arx and Kurt [25] have reported on guided bone regeneration with autogenous bone grafts harvested intraorally from the mandible covered with titanium mesh, which was rigidly affixed with microscrews to the residual jaw bone. In total, 20 implants were placed in 15 patients. Height of implant exposure (mean 6.5 mm), dehiscences (80%) or fenestrations (20%), and graft height (mean 6.2 mm) were measured [25]. After 6 months, the titanium mesh and microscrews were removed and bone regeneration was assessed [25]. The mean height of the integrated bone graft was 5.8 mm, corresponding to a mean bone fill of 93.5% [25]. The postoperative healing was overall excellent with only one site developing a soft tissue dehiscence with subsequent mesh exposure (complication rate 5%) [25]. The authors demonstrated that a titanium mesh in combination with autogenous bone grafts can represent an effective regenerative procedure for peri-implant bone defects [25].

In another study of Jung and colleagues [26], ten patients with dehiscences or fenestrations at the time of implant placement were treated with a mixture of autogenous bone particulate and allograft covered and protected by a preformed titanium mesh, which was fixed directly on the implant neck. No complications were reported in the postoperative period nor in the following months [26]. Four months after placement, small biopsies were taken from the regenerated areas: these specimens demonstrated successful and satisfactory bone regeneration, with 80% vital bone, 5% fibrous marrow tissue, and 15% remaining allograft [26]. All implants were successfully in function after a period of 1 year [26]. The authors concluded that the use of preformed titanium meshes can represent a reliable treatment procedure around peri-implant alveolar bone defects: in addition, they are extremely easy to apply, by fixing them on the implant shoulder, and simple to remove [26].

In the study of Konstantinidis and colleagues [27], peri-implant dehiscences of 26 patients who were installed with 36 implants were treated with GBR, using an alloplastic calcium-phosphosilicate putty protected by either collagen membranes (27/36 implants) or titanium meshes (9/36 implants). All implants were followed for a period of 1 year and all complications were registered [27]. During the second-stage surgery for the removal of the titanium membranes, the mean bone gain accounted to 3.23 (±2.04 mm). Almost 75% of the peri-implant defects achieved complete regeneration [27]. No complications were reported. A negative correlation was found between patient age and complete coverage of the peri-implant defect [27]. The overall implant survival rate was 97.2% at 1 year; therefore the authors concluded that the use of an alloplast in combination with either a collagen membrane or a titanium mesh can be considered a successful treatment option in case of peri-implant dehiscences of small or medium entity [27].

In our present study, the alveolar ridge reconstruction with titanium meshes and simultaneous implant placement has proved to be a reliable and effective treatment, with an average horizontal bone gain of 3.67 mm (±0.89).

This is in accordance with the contemporary scientific literature [15, 17, 18, 25–27], which reported that GBR with titanium membranes represent a predictable technique for horizontal bone regeneration and the treatment of small- and medium-sized defects around dental implants.

As reported in different systematic reviews [12, 14, 15, 18], the ideal membrane should possess the following characteristics: biocompatibility, ability to prevent the penetration of unwanted cell lines and to maintain its space, and ease of clinical handling.

The titanium meshes used in the present study meet almost all these requirements: in fact, they are biocompatible, they are efficiently integrated with the tissue, and they can effectively prevent the colonization of the site by connective tissue. In addition, they have excellent space maintenance capabilities and they are easy to use. A membrane, in fact, should be sufficiently stiff to be able to counteract the pressure exerted by external forces (such as tensions within the surgical flap and muscular tensions), but at the same time quite malleable/easy to be adapted to the defect site [12, 14, 15, 18]. The titanium meshes used here ensure excellent mechanical properties: in fact, they are able to preserve the space effectively and to contain the regenerative material (be it bone or particulate biomaterial) with great efficiency, preventing the collapse of the overlying soft tissue, or the compression generated by the same, that could determine the dispersion of the particulate during healing. Not least, they are easy to handle and can be easily adapted to the site and fixed directly to the implant, allowing the surgeon to sculpt the contours of the alveolar tissue to be regenerated. The ease of use is a key factor, since the easier is the application and adaptation of the membrane, the greater are the chances of success of regenerative therapy [15, 18, 26]. In this context, the possibility to have membranes of different sizes and shapes can be extremely helpful for the surgeon. The titanium meshes used in the present study are available in 9 different configurations, characterized by different size and shape: this helps the clinician to graft different sites (anterior and posterior sites), as alveolar bone has different widths according to locations. In fact, for incisors and cuspids, “narrow” membranes can be used, which have 4.5 mm buccal horizontal extension from the center of fixture; for premolars, “regular” membranes, which have 5.5 mm buccal extension, can be selected. For molars, a wider membrane (6.5 mm buccal horizontal extension) can be used, particularly with immediate placement cases with wall defects; these wider membranes have also a palatal/lingual extension to cover palatal/lingual wall defects.

From the analysis of the current literature, the biological complications emerge as the main problem occurring with titanium membranes, both in the immediate postoperative and in the following months [12, 14, 15, 18–27]. Our present work appears to confirm, at least in part, the evidence emerging from the literature [12, 14, 15, 18].

In this retrospective work on 25 patients, the most frequent complication, which occurred in 12 patients (12/25: 48%), was represented by the postoperative edema; the second complication was represented by postoperative pain or discomfort, which occurred in 10 patients (10/25: 40%). Both of these complications were classified as early complications; however, they were minor in nature as they could be easily managed with anti-inflammatory drugs, resolving already during the first week. The third complication per incidence (6/25: 24%) was instead represented by the exposure of the titanium mesh. The exposure of the titanium mesh is certainly one of the most insidious complications to handle, as reported in the literature [12, 14, 15, 18]; in fact, it can cause the failure of the regenerative technique. In our work, in 4 patients, this complication was managed with success and did not give consequences; in 2 patients it instead determined the infection of the graft, with the necessity of early removal of the titanium membrane. One of these two patients lost part of the graft, while the other lost the entire graft and the fixture. The implant survival at 1 year from the placement of the final restoration was high, with only one lost implant placed out of 40 (implant-based survival 97.5%). No prosthetic complications were registered, either mechanical or technical. The implants used in this study, in fact, present a conical connection (10°) combined with an internal hexagon, characterized by high mechanical stability [28–30]. The conical implant-abutment connections can guarantee high stability, as demonstrated by several recent works [32–34]. In addition, these implants possess an integrated platform switching [29, 30]; this is useful to maintain and preserve the tissue volumes, as previously reported [35–37]; accordingly, a minimal bone resorption was found around the implants, with a mean overall peri-implant marginal bone loss of 0.40 mm (±0.20) 4 months after the implant placement; this bone loss increased to 0.43 mm (±0.15) at the 1-year follow-up control.

Our present study has limits. First, although it is based on data collected from different centers (where surgeons have worked under the same surgical and prosthetic protocols), it is retrospective: retrospective studies are not the best solution to investigate clinical issues and certainly have a lower value than prospective studies. For this reason, further prospective clinical studies or even better, randomized controlled trials will be needed to confirm our present positive outcomes. Second, our present work is based on a limited number of patients (and implants), and the implants here were followed up for a short time (1 year). Therefore, further long-term studies on a larger sample of patients will be needed to evaluate the efficacy of the present treatment and the reliability of these new titanium meshes for bone regeneration of small- and medium-sized peri-implant bone defects.

## 5. Conclusions

In the present retrospective multicenter study, the authors have reported on guided bone regeneration with titanium meshes and simultaneous implant placement. In particular, a new type of titanium mesh that can be fixed directly on the fixture has been used for bone regeneration of small- and medium-sized peri-implant bone defects. Overall, the horizontal ridge reconstruction with titanium meshes positioned simultaneously with dental implants achieved predictable satisfactory results. In fact, after the removal of the titanium meshes, the CBCT evaluation revealed a mean horizontal bone augmentation of 3.67 mm (±0.89). Mild postoperative edema (48%) and pain/discomfort (40%) after surgery were the most frequent biological complications encountered, but these early complications were completely resolved within one week after surgery. Titanium mesh exposure occurred in 6 patients (24%): one of these patients suffered partial loss of the graft and another complete graft loss and implant failure. After 1 year from implant placement, an overall satisfactory implant survival rate of 97.5% (implant-based) and a limited mean peri-implant marginal bone loss of 0.43 mm (±0.15) were found. The present positive outcomes can be considered encouraging but must be confirmed by further long-term controlled studies on a larger sample of patients.

## Figures and Tables

**Table 1:** Patient-related information.

	N° patients (%)	*p*–value^*∗*^
Overall	25 (100%)	
*Gender*		
Males	15 (60%)	0.3173
Females	10 (40%)	
*Age at surgery*		
43–51	11 (44%)	0.4677
52–60	8 (32%)	
61–69	6 (24%)	
*Smoking habit*		
Yes	8 (32%)	0.0719
No	17 (68%)	
*History of periodontal disease*		
Yes	7 (28%)	0.0278
No	18 (72%)	

^*∗*^Chi-square test.

**Table 2:** Implant-related information.

	N° implants (%)	*p*–value^*∗*^
Overall	40 (100%)	
*Site*		
Maxilla	32 (80%)	0.0001
Mandible	8 (20%)	
*Position*		
Incisor/cuspids	12 (30%)	0.0074
Premolars	22 (55%)	
Molars	6 (15%)	
*Protocol*		
Healed ridges	31 (77.5%)	0.0005
Postextraction sockets	9 (22.5%)	
*Length*		
8.0 mm	7 (17.5%)	0.0203
10.0 mm	18 (45%)	
11.5 mm	10 (25%)	
13.0 mm	5 (12.5%)	
*Diameter*		
3.5 mm	19 (47.5%)	0.0655
4.0 mm	14 (35%)	
4.5 mm	7 (17.5%)	
*Type* *of* *prosthesis* ^*∗∗*^		
SCs	17 (43.6%)	0.0225
FPPs (2 units)	12 (30.8%)	
FPPs (3 units)	6 (15.4%)	
FPPs (4 units)	4 (10.2%)	

^*∗*^Chi-square test test.

^*∗∗*^Calculated on the 39 surviving implants.

**Table 3:** Peri-implant marginal bone loss between groups of implants at different time periods, in mm (implant level).

	Baseline, 4 months	Baseline, 1 year
	*N* ^*∗*^; mean (SD); median; CI 95%	*N* ^*∗*^; mean (SD); median; CI 95%
Overall	39; 0.40 (±0.20); 0.35; 0.34–0.46	39; 0.43 (±0.15); 0.44; 0.39–0.47
Healed sites	30; 0.42 (±0.21); 0.36; 0.35–0.49	30; 0.43 (±0.15); 0.44; 0.38–0.48
Extraction sockets	9; 0.35 (±0.17); 0.34; 0.24–0.46	9; 0.41 (±0.17); 0.44; 0.30–0.52

*N* ^*∗*^ = number of the surviving implants.
